# Microsatellite Analyses of Blacktip Reef Sharks (*Carcharhinus melanopterus*) in a Fragmented Environment Show Structured Clusters

**DOI:** 10.1371/journal.pone.0061067

**Published:** 2013-04-09

**Authors:** Thomas Vignaud, Eric Clua, Johann Mourier, Jeffrey Maynard, Serge Planes

**Affiliations:** 1 Laboratoire d'Excellence «CORAIL» USR 3278 CNRS – EPHE, CRIOBE, Papetoai, Moorea, Polynésie Française; 2 Direction Régionale Recherche et Technologie, French Ministry of Agriculture and Fisheries, Paris, France; 3 Center for Marine Science, CREST Research Park of UNCW, Wilmington, North Carolina, United States of America; Monash University, Australia

## Abstract

The population dynamics of shark species are generally poorly described because highly mobile marine life is challenging to investigate. Here we investigate the genetic population structure of the blacktip reef shark (*Carcharhinus melanopterus*) in French Polynesia. Five demes were sampled from five islands with different inter-island distances (50–1500 km). Whether dispersal occurs between islands frequently enough to prevent moderate genetic structure is unknown. We used 11 microsatellites loci from 165 individuals and a strong genetic structure was found among demes with both F-statistics and Bayesian approaches. This differentiation is correlated with the geographic distance between islands. It is likely that the genetic structure seen is the result of all or some combination of the following: low gene flow, time since divergence, small effective population sizes, and the standard issues with the extent to which mutation models actually fit reality. We suggest low levels of gene flow as at least a partial explanation of the level of genetic structure seen among the sampled blacktip demes. This explanation is consistent with the ecological traits of blacktip reef sharks, and that the suitable habitat for blacktips in French Polynesia is highly fragmented. Evidence for spatial genetic structure of the blacktip demes we studied highlights that similar species may have populations with as yet undetected or underestimated structure. Shark biology and the market for their fins make them highly vulnerable and many species are in rapid decline. Our results add weight to the case that total bans on shark fishing are a better conservation approach for sharks than marine protected area networks.

## Introduction

Sharks have been considered keystone species driving the evolution of marine ecosystems [1], [2]. It is a concern therefore that their biological characteristics (e.g., low fecundity, late maturity, long gestation periods) mean shark populations are highly vulnerable and sensitive to overfishing and habitat degradation. Declining populations of sharks and other top predators from marine ecosystems is likely to have cascading effects on trophic structure and ecosystem dynamics [3]. Shark populations around the world have already suffered drastic declines, ranging from 50 to more than a 90% reduction in stocks, depending on species and area, and resulting in 34% of the oceanic species facing extinction [4]. Estimates indicate that even in recent years several dozen million sharks have been killed every year [5]. These alarming figures and media attention have raised awareness of the practice of shark finning and protecting sharks has become a worldwide conservation priority.

Effective conservation planning for any organism can partly rely on defining the spatial extent of populations, the levels of exchange (connectivity) among them and their distribution in space [6], [7]. Sharks are usually described as highly mobile species that often have large home ranges. As a consequence conventional marine protected areas (MPAs) and MPA networks are not likely to be effective in protecting shark populations [8] since home ranges are often larger than typical MPA sizes. Correctly estimating the connectivity between demes or populations is difficult and creates issues for and raises the uncertainty of the population models used to plan MPA networks [9]. Shark ecological traits generally differ significantly from those of most marine teleosts. For instance, sharks do not have a larval phase and for most species dispersal occurs only during adulthood [10]. Also, effective population sizes are much smaller for sharks relative to those of small reef fish species. These characteristics have important implications for the dynamics and evolution of shark populations.

Several studies that have found significant genetic heterogeneity among populations of shark species have included samples between oceans (e.g. [11]–[13]) leading to the suggestion of genetic homogeneity within ocean basins except in rare occasions (e.g. [14], [15]). When genetic heterogeneity has been found on smaller scales for some shark populations, evidence for explanations like a bottleneck and/or recent colonization has been presented, or the subject species is slow-moving (e.g. [14]–[22]). Here we assess the level of genetic structure among demes of blacktip reef sharks in the island chains of French Polynesia in the south-central Pacific Ocean. Given the literature, it is hard to hypothesize about blacktip reef shark dispersal and gene exchange. They are small coastal sharks commonly residing within coastal habitats that can be separated from other suitable habitat by hundreds of km, which could suggest limited dispersal. However, they have colonized remote islands, are fast active-swimming species, and a recent study has shown that blacktip reef shark females in French Polynesia will cross open water between islands as much as 50 km apart [23]. Whether dispersal occurs in the fragmented environment of French Polynesia frequently enough to prevent moderate genetic structure is unknown.

French Polynesia is a prime example of a fragmented marine environment for a shark species known to inhabit coastlines; there are 130 islands in French Polynesia spread across 2,500,000 km^2^ of ocean [24]. Coral reef fish communities and blacktip reef sharks are concentrated and aggregated around islands separated by large bodies of open deep water. The open water separating the islands may act as at least a partial barrier to dispersal; the waters between the islands can be >1000 m deep and some islands are separated by >100 km. Blacktip reef sharks are viviparous and produce a few (2–5) pups generally once a year. The gestation period is about 10–11 months, and it is likely that sexual maturity is not reached before 4–5 years old [25], [26]. They are easy to catch in shallow waters either in the lagoon or outside the barrier reef. Shark populations have been over-fished in many coastal areas [6], [27]–[30] but fishing pressure on sharks is still relatively low in French Polynesia. Human population numbers are low, the local culture favors sharks and laws have recently been passed to protect sharks. Shark population dynamics are thus likely to be as close to natural as possible. Some sharks are killed in remote islands to prevent damage to fishing gear and traps but direct human pressure on blacktip reef sharks in French Polynesia is low.

We tested for the presence of genetic structure among 5 shark demes from the reef and coastal habitat surrounding 5 different islands with varying inter-island distances (50 to 1500 km). We use the word ‘deme’ here to define a group of individuals that have been sampled around the same island and because these may not be populations per se.

## Methods

### Study area and sampling

**Figure 1:**
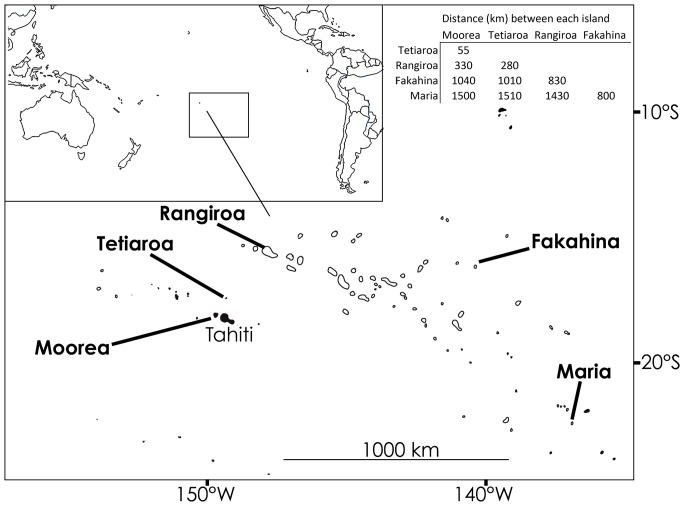
Map of French Polynesia showing the 5 sample locations. Distances (in km) between the sample locations are shown in the matrix top right.

Locations of sampling sites in French Polynesia are detailed in **Figure 1**. A total of 165 individuals were sampled from 5 locations: Moorea (high island, N = 38), Tetiaroa (small semi-closed atoll, N = 12), Rangiroa (large open atoll, N = 35), Fakahina (small closed atoll, N = 41) and Maria (small closed atoll, N = 39). These locations ensure a variety of distances between sampling sites. In each location, sharks were fished with barbless circled hooks and metal lines from the shore or from a boat. The animals were sized, sexed, and a small tissue sample was taken from either a dorsal or anal fin. Each shark was then released in good condition. Fin clip samples were conserved in 90% Ethanol.

### Laboratory procedures

DNA samples were extracted following the PURAGENE™ DNA Purification Kit procedure for 5–10 mg fresh tissue. PCR was conducted using 4 multiplexes for 11 microsatellite loci, and following QIAGEN® Multiplexes PCR Kit procedure. The loci characteristics and thermal cycling conditions used are described in **Table S1** and were taken and adapted from [31]–[33] and optimized for the target species and multiplex PCR. PCR products were analyzed using a capillary electrophoresis sequencing Beckman CEQ™8000, and the fragment sizes were manually read using the program CEQ™8000.

### Data analyses

A first set of analyses was computed to test the quality of the 11 markers. Microcheckerv2.2.3 [34] was used to check potential genotyping errors and null allele(s). GenAlEx [35] was used to test for the Hardy-Weinberg equilibrium for each locus in the entire dataset, and for each locus in each deme. GenAlEx was also used to compute general loci information such as allelic richness and private alleles.

We used one genotypic clustering approach and two allele-based statistics approaches (*F*_ST_ and *R*_ST_) [36]–[39] to measure the genetic structure between the 5 demes. The clustering approach used is the one implemented in Adegenet [40] for R [41], based on a Bayesian algorithm for the Discriminant Analysis of Principal Component (DAPC) [42]. The optimal number of principal components chosen for the analysis was of 21, following indications of alpha scores [42], [43].

Pairwise *F*_ST_ and *R*_ST_ values were calculated using the AMOVA method implemented in GenAlEx, in order to measure the genetic differences between demes. These values were also calculated for each locus to help understand potential discrepancies between the global pairwise *F*_ST_ and *R*_ST_ values. Additionally, an AMOVA was performed using GenAlEx to determine the global level of differentiation among clusters.

We used ARES [44] to estimate the allelic richness for each deme and to extrapolate it to the higher number of individuals among samples, for comparison among demes (**Table S2**). We used the program Bottleneck [45] to check for bottlenecks and changes in effective population size. To inform this analysis we checked heterozygote excesses, using the 3 different models: the infinite alleles model (IAM), the stepwise-mutation model (SMM) and the two-phase microsatellite evolution model (TPM) with 70% SMM and 30% IAM.

Because of the low number of demes, the correlation between geographic distance and genetic differences was examined by jackknifing over populations, using Isolation By Distance [46]. This approach calculates the slope and intercept of the relationship between genetic distances and geographic distances using a Reduced Major Axis regression. We used the log of *F*_ST_ values and the log of geographic distances, as suggested in [47] when using distances in two dimensions.

Considering the stepwise mutation model, we used the program SPAGeDI [48] to test if stepwise-like mutations contribute to genetic differentiation, using the *R*_ST_/p*R*_ST_ test described in [49]. Loci with 3 or less alleles (Cli103, Cli2 and LS20) were excluded from the analysis. Finally, a sex-bias dispersal test was performed using GenAlEx following the method described in [49], [50], and deleting all juveniles and small sub-adults (N = 35) from the dataset.

## Results

Heterozygosity ranged from 0.077 (Cli2) to 0.865 (LS53) with a mean of 0.492 (**Table 1**). Overall, none of the loci showed significant departures from the Hardy-Weinberg equilibrium (all p-values>0.05). However, when looking at the patterns from each deme, 3 loci diverged from the Hardy-Weinberg equilibrium in the deme from Moorea (Cli107, Cli111, LS54) and 2 in the deme from Rangiroa (Cli108 and LS32). Cli2 is monomorphic in the deme from Fakahina. Within all samples (165 individuals) and the 11 microsatellites we found 79 alleles including 14 private alleles distributed in 10 of the 11 loci. Allele frequencies for each deme are ordered by size in **Table S3**, which has a histogram describing differences in allele frequency between demes. Differences in allele frequencies are used here to estimate genetic differences between demes. These raw frequencies, for each deme and each locus, are very different in some of our cases. As an example, the allele 220 in the locus LS20 is present 5.7% of the time in the Rangiroa deme but 42.4% of the time in the Fakahina deme.

**Table 1:** Expected (He) and observed (Ho) heterozygosity for each locus and each deme.

Loci name	Cli-102		Cli-107		Cli-111		Cli-103		Cli-108		Cli-2		LS-54		LS-75		LS-53		LS-32		LS-20	
Sampling location	He	Ho	He	Ho	He	Ho	He	Ho	He	Ho	He	Ho	He	Ho	He	Ho	He	Ho	He	Ho	He	Ho
Moorea N = 38	16.6	15	17.3	26	34	32	18.8	18	24.1	27	5.6	4	12.21	8	23.9	18	35	33	16.6	18	8.8	10
Rangiroa N = 35	18.1	20	15.6	20	31.8	32	14.9	13	22.4	20	2.9	3	20.1	25	18.7	21	33.3	32	12.6	13	3.8	4
Fakahina N = 41	20	25	25	30	34.4	28	5.6	6	22.8	21	0	0	16.4	19	23.4	18	33.5	34	6.5	7	20.1	14
Tetiaroa N = 12	7.1	6	7.8	10	9.5	8	5.6	8	7.2	8	1.9	2	5.2	5	7.2	8	10.5	11	5.1	6	2.7	3
Maria N = 39	9.6	7	14	17	31	32	19.7	23	20.4	24	1	1	7.5	8	8.1	7	34	31	10.3	12	17	17

### Genetic structure

The DAPC results for the 21 principal components found show a clear genetic structure for all demes.

**Figure 2:**
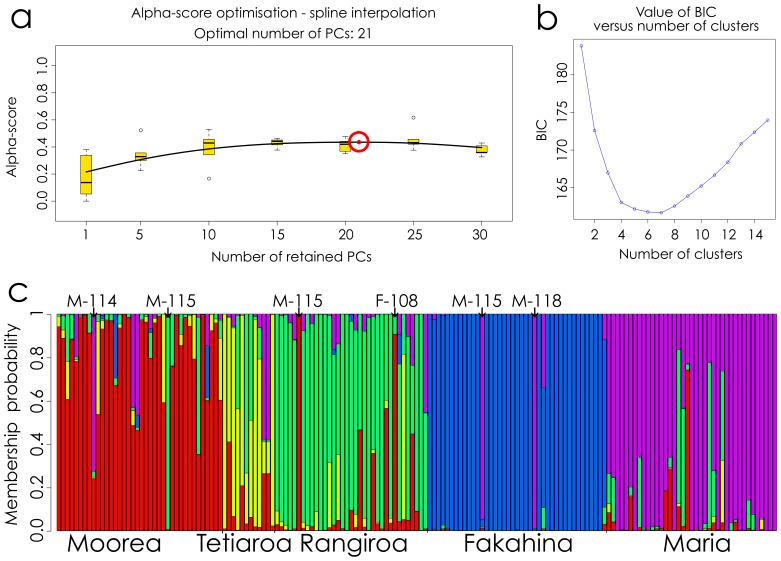
Bayesian approach results using the Discriminant Analysis of Principal Components to investigate genetic structure. The optimal number of principal components found for the analysis was 21 based on the trend in alpha scores (a). The BIC (Bayesian Information Criterion) values are shown in relation to the number of genetic clusters in (b). Each vertical bar represents an individual in the DAPC diagram (compoplot) shown as (c), and each color represents the probability of belonging to one of the genetic clusters. Some outliers have been noted on the top of the figure: M = male, F = female, followed by the total length of each shark in cm.

Overall, individuals are associated with their original deme. The membership probability is particularly strong for Fakahina as a majority of individuals are 100% associated with the Fakahina deme. A large number of unsampled demes are likely reinforcing the structure of isolated demes. However, individuals among the 4 other demes also show strong membership probability to their original deme, but with more individuals having mixed signals than it was the case with Fakahina. There is very little genetic signal from Fakahina in other demes. In contrast, Maria has mixed individuals with signal mostly from Moorea and Rangiroa. Some outliers are observed in all clusters, suggesting potential recent migrations. These results are depicted in **Figure 2**, including the sex and size of a few outliers. The outliers are mostly male around 115 cm in total length, roughly the size at which they become mature. The lowest Bayesian Information Criterion (BIC) values were between 4 and 6, although there is no clear elbow in BIC value curve.

Pairwise ***F*** **_ST_** results show high values for the Fakahina deme (*vs.* each of the other demes; ***F*** **_ST_** = 0.097, 0.098, 0.142 and 0.148) and for the Maria deme (*vs.* each of the other demes; ***F*** **_ST_** = 0.080, 0.095, 0.148 and 0.106, **Table 2**). The ***F*** **_ST_** values associated with Fakahina are higher than those associated with Maria, even though Maria is further from the other islands. The Moorea, Tetiaroa and Rangiroa demes are less differentiated among each other (***F*** **_ST_** = 0.038, 0.025 and 0.043). ***F*** **_ST_** results are highly significant for all deme pairs (p = 0.01). Fakahina *R*_ST_ values are of 0.301 versus Moorea, 0.437 versus Tetiaroa, 0.111 versus Rangiroa and 0.081 versus Maria (**Table 2**). Maria *R*_ST_ values, except for versus Fakahina, are non-significant and small (0.009, 0, 0.003). Rangiroa *R*_ST_ values versus Moorea and Tetiaroa are small (0.0041 and 0.126) but significant (p = 0.017 and 0.008). The Moorea versus Tetiaroa *R*_ST_ value is 0.054, and is non-significant (p = 0.063).

**Table 2:** Pairwise *F*_ST_ and *R*_ST_ values from GenAlEx. P-values are above diagonal.

	*F*_ST_				
	Moorea	Tetiaroa	Rangiroa	Fakahina	Maria
Moorea		0.010	0.010	0.010	0.010
Tetiaroa	0.025		0.010	0.010	0.010
Rangiroa	0.038	0.043		0.010	0.010
Fakahina	0.097	0.142	0.098		0.010
Maria	0.080	0.105	0.095	0.148	

	*R*_ST_				
	Moorea	Tetiaroa	Rangiroa	Fakahina	Maria
Moorea		0.063	0.017	0.001	0.312
Tetiaroa	0.054		0.008	0.001	0.162
Rangiroa	0.041	0.126		0.001	0.511
Fakahina	0.301	0.437	0.111		0.001
Maria	0.009	0.000	0.003	0.081	

**Figure 3:**
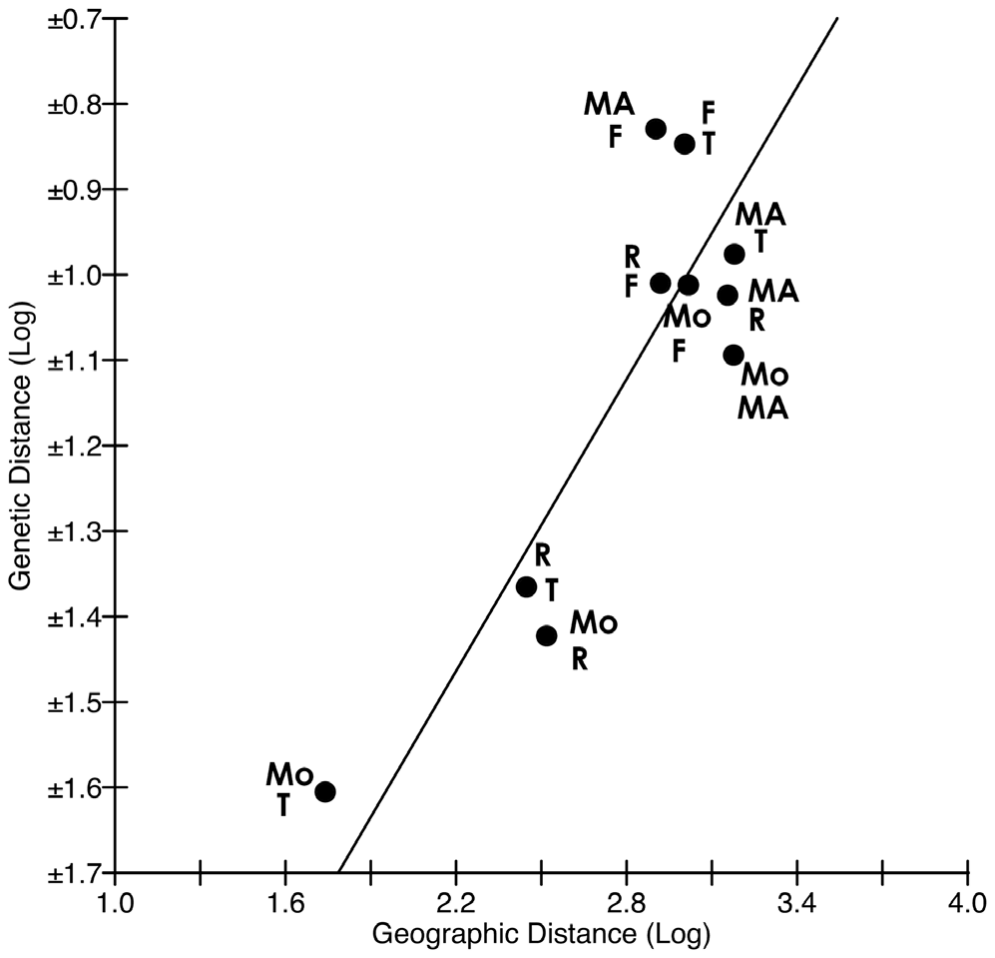
Isolation by Distance graph comparing geographic and genetic distance. Jackknifing slope over the 5 demes is 0.3654±0.275 (SE). The data points denote two-location comparisons: Mo = Moorea, R = Rangiroa, F = Fakahina, T = Tetiaroa and Ma = Maria (R2 = 0.739).

AMOVA for 3 geographic clusters (Fakahina vs. Maria vs. others) indicated that 8% of the variation was caused by variation among geographic clusters, 38% by the variation within individuals and 54% by the variation among individuals. When an AMOVA was performed for either 4 or 5 groups, the ‘among populations’ value was 7%. The Isolation by Distance shows that genetic and geographic differences are correlated (R^2^ = 0.739); the slope after jackknifing over the 5 demes (0.3654) has a large standard error (0.275, **Figure 3**).

### General population information

As a proxy of effective population size, we estimated values of allelic richness for each deme. To compare these values we had to restrict (Fakahina)/extrapolate (Moorea, Tetiaroa, Rangiroa, Maria) the number of samples used for the analysis to 40. The estimated values of allelic richness ranged from 68 for Rangiroa to 44 for Fakahina and Maria, 57 for Moorea and 53 for Tetiaroa. Despite these differences in allelic richness, no evidence of a bottleneck was found, using the IAM, SMM and TPM (all>0.05). The microsatellite allele frequency distribution was L-shaped for each locality, indicating that these were all at mutation-drift equilibrium for the loci used. The *R*_ST_/p*R*_ST_ results on the 8 loci with more than 3 alleles are significant for Moorea-Fakahina (P(one sided test, H1: obs<exp) = 0.984) and for Tetiaroa-Fakahina (P(one sided test, H1: obs<exp) = 0.977) (**Table 3**). There are large differences though when looking at the results for each locus, and the high variance in the results for Tetiaroa may be due to a low sample size.

**Table 3:** *R*_ST_/p*R*_ST_ results from SPAGeDI.

		ALL LOCI	Cli102	Cli107	Cli111	Cli108	LS54	LS75	LS53	LS32	ALL LOCI	ALL LOCI
Pairwise locations		P (1-sided test, H1: obs<exp)									Obs val	Mean permut val
Moorea	Tetiaroa	0.787	0.334	1	0.501	0.758	0.672	0.665	0.898	0.335	0.054	0.025
Moorea	Rangiroa	0.828	1	0.747	0.05	0.901	0.497	0.247	0.89	1	0.04	0.019
Moorea	Fakahina	0.984	1	0.326	0.742	0.493	0.366	1	0.98	1	0.3	0.08
Moorea	Maria	0.52	0.346	1	0.736	0.181	0.711	0.336	0.493	1	0.049	0.06
Tetiaroa	Rangiroa	0.909	0.83	0.084	0.519	0.99	0.367	0.176	0.909	1	0.125	0.039
Tetiaroa	Fakahina	0.977	0.835	0.41	0.946	0.677	0.744	1	0.955	1	0.437	0.128
Tetiaroa	Maria	0.603	0.169	0.332	0.283	0.093	1	0.342	0.695	1	0.104	0.102
Rangiroa	Fakahina	0.717	0.329	0.397	0.827	0.849	0.763	1	0.679	0.242	0.11	0.084
Rangiroa	Maria	0.366	1	0.161	0.766	0.489	0.753	0.246	0.275	0.083	0.028	0.057
Fakahina	Maria	0.904	1	0.412	0.996	0.616	0.698	0.665	0.797	1	0.248	0.103

Significance values from all loci are followed by the values for each of the 8 loci with more than 3 alleles. *R*_ST_ and p*R*_ST_ values for all loci are also indicated.

## Discussion

In combination, the *F*_ST_ and *R*_ST_ estimators can provide valuable information about the genetic structure of populations with some provisos [51]. These measures have different qualities and work under different assumptions, none of which are fully known or constant in all cases. One of the reasons for this involves the highly versatile behavior of microsatellites. Microsatellites are not strictly in the Infinite Allele Model (IAM) or the Stepwise Mutation Model (SMM), and many have varying mutation rates. Other reasons to carefully interpret *F*_ST_ and *R*_ST_ results include the presence of evolutionary forces like genetic drift [52] or ghost populations [53].

Even given the above, in the case presented here pairwise *F*_ST_ values are high, suggesting strong genetic differences among demes. This is especially true for the Fakahina and Maria demes, which are further from Moorea, Tetiaroa and Rangiroa than any of these are from each other. The Isolation by Distance analysis shows that genetic differentiation (observed from *F*_ST_ values) mostly increases with the distance separating demes. With only 5 sampled demes, it is hard to make general conclusions based on the Isolation by Distance results. Pairwise *F*_ST_ values are slightly higher though for Fakahina than for Maria, even though Maria is furthest the other islands sampled. This could be due to differences between these islands in the degree of isolation. The island closest to Fakahina is more than 200 km away while 4 islands in the Tuamotu chain are within 100 km of Maria. The proximity of islands in the Tuamotu chain to Maria is likely promoting some gene flow. The effective population size estimates of Fakahina and Maria are exactly the same based on allelic richness and so probably do not affect the comparison of their *F*_ST_ values.

*F*_ST_ values are known to underestimate genetic differentiation in the case of strong genetic structure [53]. It is thus possible to tentatively conclude that there is poor gene flow between Fakahina and Maria, and between the other sampled demes and Fakahina or Maria, and a moderated gene flow in the cluster Moorea-Tetiaroa-Rangiroa. The Bayesian approach results visually strongly suggest genetic differentiation, adding further support to this conclusion (see **Figure 2**). These results probably indicate a limited gene flow between the investigated blacktip shark demes. The Bayesian approach can result in the detection of a number of “populations”, usually written as ‘K’, [54]. We do not present a result for K here because it does not capture the more complex ecological reality of our case. Isolation by Distance and the presence of numerous unsampled demes may affect clustering approaches. These effects can create groups that do not necessarily exist if the sampled groups represent only a few points along a potentially graded genetic continuum. Even so, high levels of gene flow rapidly overwhelm the influence of other factors, leading to less structured demes than we see here. This does not mean that any of the demes, even Fakahina and Maria, are completely isolated as they are probably mixed with closer unsampled demes. The level of genetic structure seen in the study demes may also be influenced by small population sizes associated with genetic drift.

The Fakahina deme appears more differentiated when using the *R*_ST_, potentially indicating that the gene flow is not sufficient to counter the effect of a higher level of genetic divergence. The only significant *R*_ST_/p*R*_ST_ results are associated with Fakahina. Variance is high for the results among loci but this result is consistent with the particular geographic isolation of Fakahina. Migration rates are unlikely to be sufficient at Fakahina to keep allele sizes from diverging from the other demes. When these new microsatellite alleles appear in a deme, it means that gene flow has been especially low for many generations.

The patterns are not consistent though – there are never more than 4 loci significant for the same pair of demes. As with the *F*_ST_ results this suggests structuration by genetic drift with some level of gene flow. Surprisingly, *R*_ST_ values associated with Maria (except for Maria-Fakahina) are small and non-significant. The small *R*_ST_ values associated with Maria deme probably come from loci that do not fit the SMM behind the *R*_ST_ calculation. We suggest the *R*_ST_ values between demes are misleading in comparison to the results of *F*_ST_ value comparisons. Our case for this is that the *F*_ST_ values, the results of the Bayesian approach and the allele distribution (see **Table S3**) all suggest that Maria deme is strongly differentiated from any other deme.

Tests for sex-biased dispersal seem to hint at a pattern of male-based dispersal (non-significant result) but the sample sizes are likely too low to detect a pattern. Most of the outliers found in the clustering analysis are indeed males, supporting the suggestion that male-based dispersal is a life-history trait of these populations. This is aligned with the findings of other similar studies, like that of *Carcharhinus limbatus* in [55], and reviewed in [56]. Females also have the capacity to move from one island to another and have been shown to cross open waters in French Polynesia to give birth in the waters near islands where they may have been born [23]. Consistently giving birth in environments known to be suitable and safe could be part of a powerful evolutionary process. This could explain the lower detection of female dispersal in this study as the range of females ensures they can come back to a nursery area regularly (every year in some case) to give birth. It is unlikely though that philopatric behavior would always occur, or would have prevented the species from colonizing new areas.

Understanding the complex process driving genetic structure can be tricky, and results must be treated with caution (reviews and examples in [52], [57]–[59]). It is likely that the genetic structure seen for the blacktip demes is the result of all or some combination of the following: low gene flow, time since divergence, small effective population sizes, and the standard issues with the extent to which mutation models actually fit reality. We suggest low levels of gene flow as at least a partial explanation of the level of genetic structure seen among the sampled blacktip demes. This explanation is consistent with the ecological traits of blacktip reef sharks, and that the suitable habitat in French Polynesia is highly fragmented.

Evidence for genetic structuration of the blacktip demes we studied highlights that similar species may have populations with as yet undetected or underestimated structure. The diversity of genes tested and techniques used in the literature make the possible comparisons with other shark studies irrelevant. It is noteworthy though that genetic subdivision has been found when investigating active swimming coastal shark species. These include *Carcharhinus sorrah* in the Indo-Australian archipelago [14], and *Carcharhinus limbatus* in the northwestern Atlantic Ocean [15]. Once evidence for structure is found, logical follow-on research involves helping understand the causes. Sequencing more genes and analyzing the overall genetics with coalescent models represents planned future work with blacktips in French Polynesia. Such research may increase our understanding of the relative influence of levels of gene exchange, genetic drift and divergence time on the levels of genetic structure we document here.

Finding moderate levels of genetic structure in the blacktip reef shark demes we studied has implications for management and conservation planning. Both the adults (as reproducers and potential dispersers) and juveniles/sub-adults (as future adults) need to be protected to ensure shark populations are self-sustaining. Range sizes for sharks are difficult to assess but are highly likely to be larger than the proposed areas management agencies and conservationists plan to have in a marine protected area (MPA) network. Further, shark populations are naturally small, at least in fragmented environments. Protecting large areas will not result in the population and biomass increases seen in teleost fish protected in MPAs. For these reasons it is important to consider shark populations in conservation planning but MPAs are not likely to be the best solution for protecting sharks. The management ‘best practice’ approach to protecting most shark populations is a total ban of shark fishing everywhere this is possible. The Bahamas, French Polynesia, and Palau are examples of locations where such a ban has already been implemented. These nation-wide regulations with other recently implemented shark sanctuaries represent steps in the right direction. Other management actions like MPAs remain critical and can have positive effects on shark populations but are insufficient to avoid the severe declines caused by shark fishing.

## Supporting Information

Table S1 Locus description with sequence, repeat type and hybridization temperatures. All ‘Cli’ are taken from [31]. LS20, LS32 and LS53 are taken from [32]. LS54 and LS75 are taken from [33].
(TIF)

Table S2 Extrapoled allelic richness and means. Tetiaroa was excluded because of a low number of samples (12).
(TIF)

Table S3 Allele frequencies for each deme ordered by size (left) and presented as histograms.
(TIF)
