# Supplementary figures and images for: Microsatellite Analyses of Blacktip Reef Sharks (Carcharhinus melanopterus) in a Fragmented Environment Show Structured Clusters
Source: PLoS One. 2013 Apr 9;8(4):e61067. doi: 10.1371/journal.pone.0061067 (PMC3622011; doi:10.1371/journal.pone.0061067)

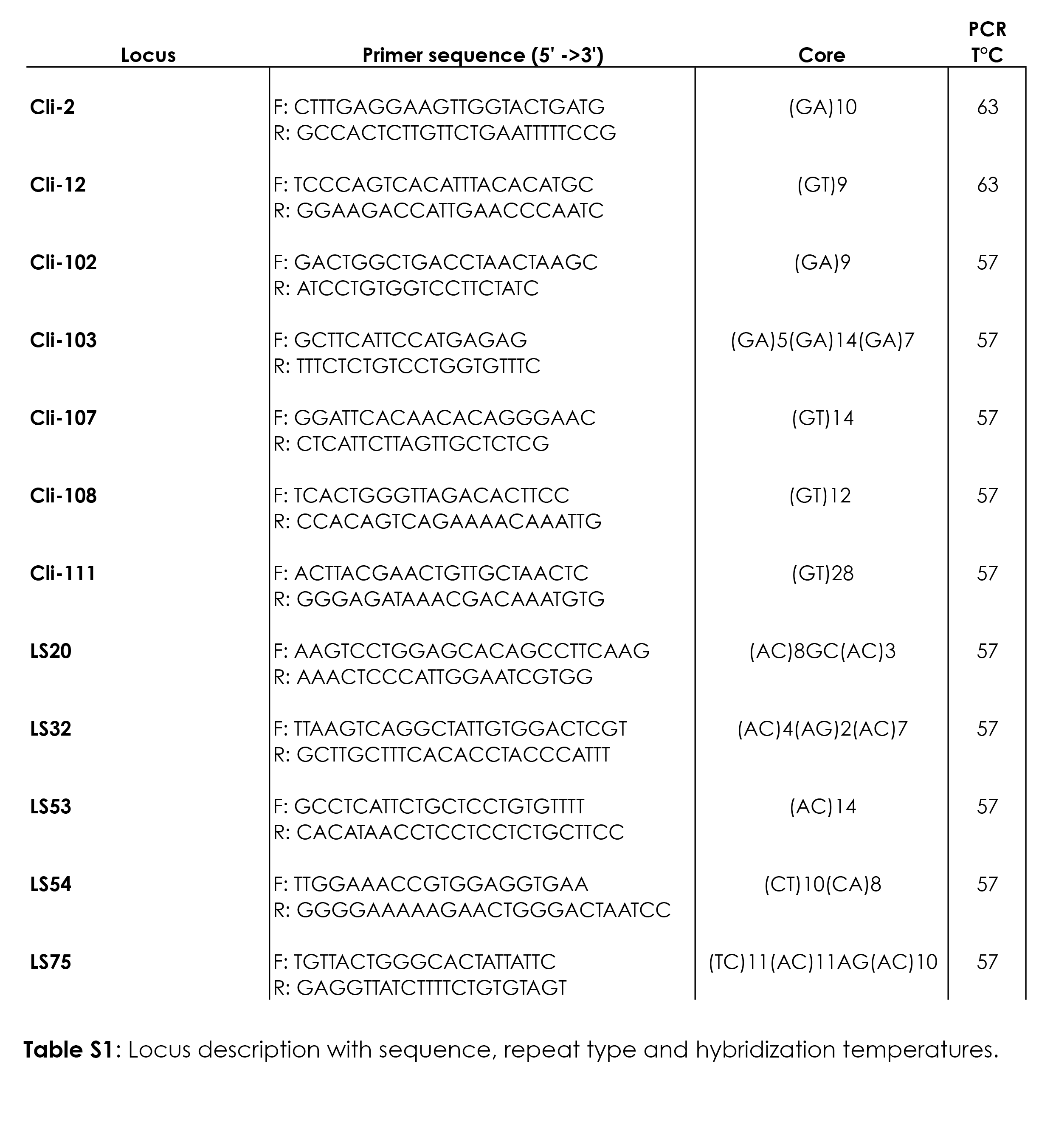

Supplement: Table S1 — Locus description with sequence, repeat type and hybridization temperatures. All ‘Cli’ are taken from [31]. LS20, LS32 and LS53 are taken from [32]. LS54 and LS75 are taken from [33]. (TIF) [file pone.0061067.s001.tif]

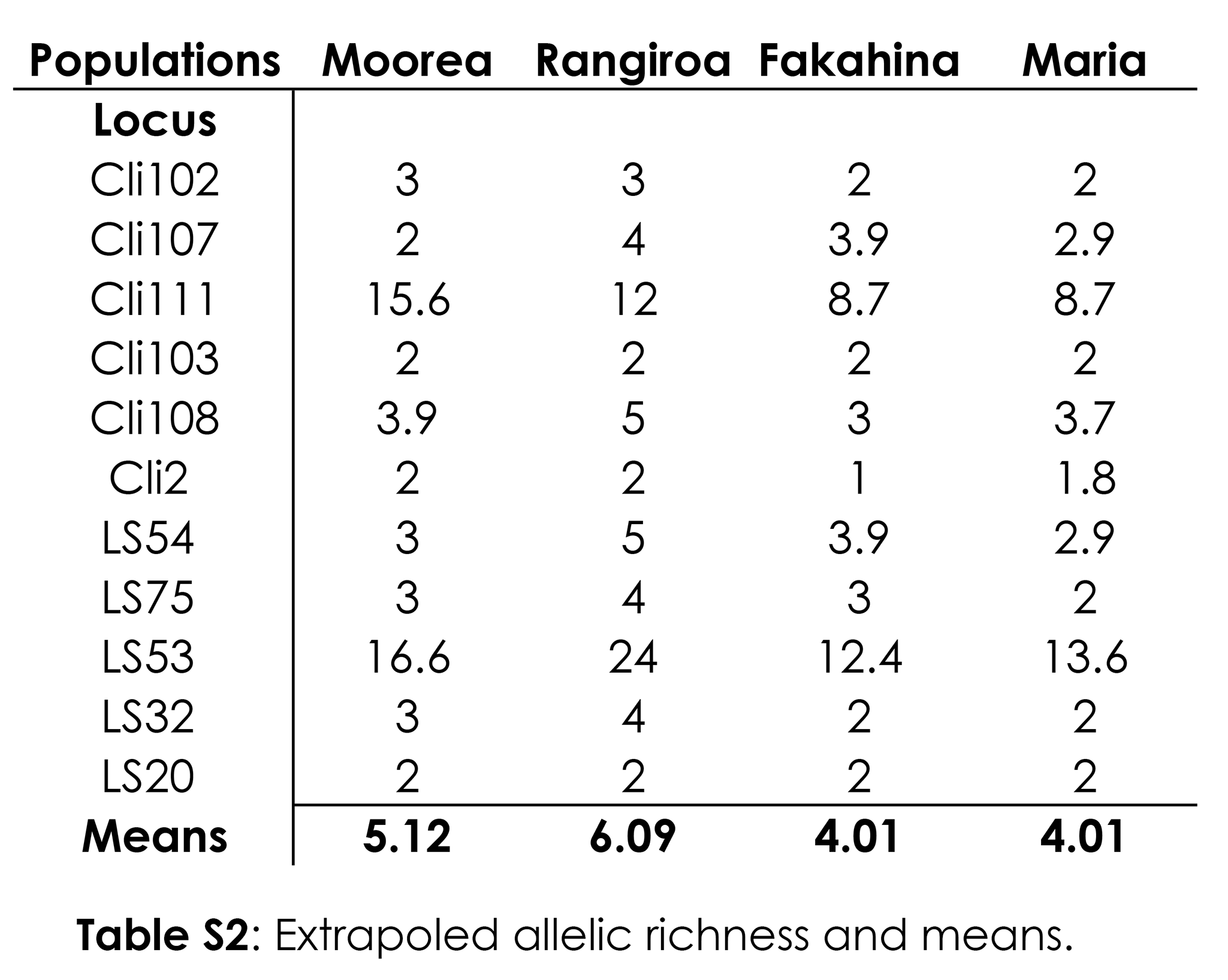

Supplement: Table S2 — Extrapoled allelic richness and means. Tetiaroa was excluded because of a low number of samples (12). (TIF) [file pone.0061067.s002.tif]

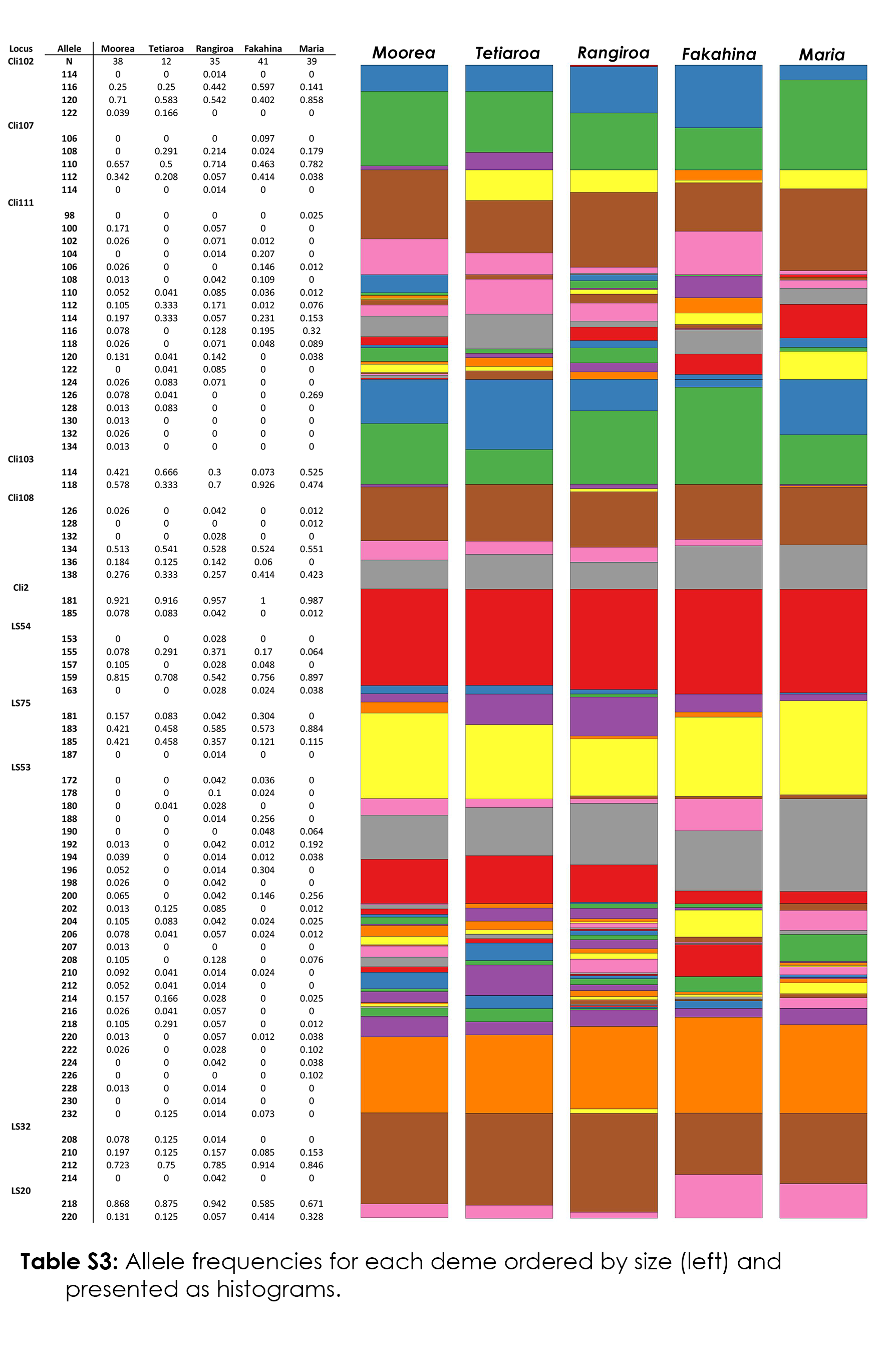

Supplement: Table S3 — Allele frequencies for each deme ordered by size (left) and presented as histograms. (TIF) [file pone.0061067.s003.tif]
